# Canine Leishmaniasis in Morocco: A Descriptive Prospective Clinical Study

**DOI:** 10.1155/2021/6304127

**Published:** 2021-09-06

**Authors:** Houda Idrissi, Maryam Hakkour, Luc Duchateau, Renato Zanatta, Malika Kachani, Rahma Azrib, Sylvie Daminet, Faouzi Kichou, Sabrine El Asatey, Noureddine Tazi, Hamid Sahibi, Sarah El Hamiani Khatat

**Affiliations:** ^1^Department of Pathology and Veterinary Public Health, Hassan II Agronomy and Veterinary Institute, Rabat-Instituts, Madinat Al Irfane, PO Box 6202, Rabat, Morocco; ^2^National Reference Laboratory of Leishmaniasis, 27 Avenue Ibn Batouta, PO Box 769, Agdal, Rabat, Morocco; ^3^Laboratory of Biodiversity, Ecology and Genome, Faculty of Sciences, Mohammed V University in Rabat, Agdal, Rabat, Morocco; ^4^Biometrics Research Center, Faculty of Veterinary Medicine, Ghent University, Salisburylaan 133, 9820 Merelbeke, Belgium; ^5^Department Patologia Animale, Faculty Veterinary Medicine, Largo Paolo Braccini 2, 10095 Grugliasco (Torino), Italy; ^6^College of Veterinary Medicine, Western University of Health Sciences, 309 E. Second Street, Pomona, CA 91766-1854, USA; ^7^Department of Medicine, Surgery and Reproduction, Hassan II Agronomy and Veterinary Institute, Rabat-Instituts, Madinat Al Irfane, PO Box 6202, Rabat, Morocco; ^8^Small Animal Department, Faculty of Veterinary Medicine, Ghent University, Salisburylaan 133, 9820 Merelbeke, Belgium; ^9^Veterinary Clinic of the 1st Kennel of the Royal Armed Forces, 13000 Benslimane, Morocco

## Abstract

Canine leishmaniasis (CanL) is a zoonotic vector-borne disease that is endemic in the Mediterranean Basin including Morocco. Dogs play a major epidemiological role in this zoonosis as reservoir hosts. This study investigated the clinical manifestations of CanL in dogs naturally infected with *Leishmania infantum*. A total of 96 dogs presented to the Small Animal Clinic of the Hassan II Agronomy and Veterinary Institute (IAV Hassan II) of Rabat, Morocco, and were tested by RT-PCR and/or serology. Among them, 32 (33.3%) were positive to *Leishmania infantum* infection. The majority of the positive dogs (93.7%) came from urban areas. Most of them were male (62.5%) and purebreds (65.6%), were aged between 3 and 7 years (71.8%), and had outside activities (guarding, hunting, livestock guarding, and service activities) (71.8%) and all of them were living exclusively outdoor or had free access to the outdoor environment. Lymphadenomegaly (81.2%), dermatological disorders (65.6%) (mostly exfoliative dermatitis), weight loss (59.3%), exercise intolerance (56.2%), anorexia (28.1%), hyporexia (15.6%), and ocular lesions (28.1%) were the most frequent clinical signs and complaints recorded. Anemia and hyperproteinemia due to hyperglobulinemia were observed in 68.7% and 72.7% of the cases, respectively. These results suggest that CanL leads to various nonspecific clinical signs as described previously, making the diagnosis challenging. Since CanL is endemic in Morocco, it should be recommended to systematically test dogs displaying clinical signs compatible with this disease and to regularly screen asymptomatic at-risk dogs. It is also crucial to educate dog owners about the zoonotic aspect of the disease and to encourage intersectorial collaboration following the “One Health” concept, in order to contribute to a more effective control/prevention of human and canine leishmaniasis.

## 1. Introduction

Canine leishmaniasis (CanL) is a zoonotic vector-borne disease caused by the protozoan *Leishmania* spp. and it is transmitted by the bite of phlebotomine sand flies [1]. *Leishmania infantum* species is the main causative agent in the Mediterranean Basin [2–4]. However, *Leishmania tropica* has also been demonstrated to cause the disease in some Mediterranean countries, such as Turkey [5].

This parasitic infection is endemic in the Mediterranean Basin, where the seroprevalence ranges between 10 and 37% [6]. However, studies have shown that the seroprevalence in Morocco can be much higher than those of the rest of the Mediterranean countries (8.6% [7], 24.5% [8], and 87.8% [6] in the northern, central, and southern regions of the country, respectively).

Dogs constitute the main domestic reservoir for human visceral leishmaniasis (VL) caused by *L. infantum* and play a key role in the transmission to humans [9]. Studies carried out on cutaneous leishmaniasis (CL) in the Maghreb countries isolated *L. infantum* from dogs too, suggesting them as a possible reservoir of this disease as well [10].

A total of 2,298 cases of human VL and 78,001 cases of CL have been reported in Morocco between 1997 and 2018. However, recent molecular investigations have shown that CL due to *L. infantum* is beginning to have a wider distribution, especially in the northern region of the kingdom where the visceral form dominates [10].

In Morocco, dog ownership has increased in the past years. Moreover, loyalty of dogs and their usefulness in various activities (hunting, guarding, service, etc.) make them necessary in the social life of the Moroccan population, especially in rural areas. Various studies estimated an average of 1.7 to 3.4 dogs per household with up to 8 dogs per household and a percentage of 66.3% to 83% of households with at least one dog [11–13]. In addition to owned dogs, stray dogs are still a major problem in Morocco. They seem to represent 10% of the total dog population [14] and participate in the transmission of some zoonotic diseases including leishmaniasis.

Generally, CanL causes a wide variety of clinical signs ranging from an asymptomatic form to a severe and fatal disease if proper diagnosis and therapy are not achieved [15]. It seems to be the most studied vector-borne disease in dogs in Morocco [7, 16–19]. However, to our knowledge, this is the first study that aimed to examine, describe, and discuss clinical signs of CanL in Moroccan dogs brought to a veterinary hospital, and coming mostly from urban areas where CanL has not been studied before.

Due to the increasing prevalence of VL, the growing canine population, the close relationship between the human population and dogs, and the lack of recent studies on CanL in Morocco, we proposed describing the clinical signs associated with the disease in dogs that presented to the Small Animal Clinic of IAV Hassan II, Rabat, Morocco. In addition, necropsy of a *Leishmania*-positive euthanized dog enabled us to provide an illustrated description of the macroscopic and microscopic lesions.

## 2. Materials and Methods

### 2.1. Period and Location

The study took place from March 2018 to December 2019 at the Small Animal Clinic of IAV Hassan II. The hospital is located in Rabat, the capital city of Morocco. It received first-line and referred cases. Each year, an average of 1085 dogs and 493 cats are admitted to the hospital, coming mostly from across Rabat and less frequently beyond.

### 2.2. Study Population

A total of 96 dogs presented to the Small Animal Clinic of IAV Hassan II, Rabat, Morocco, presenting a history (living in endemic areas, spending most of the time outside the house, in close contact with positive animals, at-risk activity such as hunting, or any frequent outdoor activity) or clinical signs (decreased appetite, exercise intolerance, weight loss, lymphadenomegaly, and cutaneous, ocular, gastrointestinal or orthopedic disorders that are not related to other diseases) compatible with leishmaniosis, were included in the study.

For each dog, a detailed questionnaire was filled with the owner including the signalment (breed, age, sex, and reproductive status) and history (role or activity, hometown and cities/areas of origin, outdoor access, vaccination, ectoparasite and/or endoparasite prevention, medical history, current medications, and presenting complaints). All dogs underwent a meticulous physical examination.

Oral informed consent was obtained from the owners before inclusion of the dogs in the study.

### 2.3. Sample Collection and Diagnostic Tests

For all selected dogs, a blood sample and/or fine needle aspirate of enlarged peripheral lymph nodes were/was performed. The blood samples were collected on a serum separating tube from the cephalic or jugular vein. All enlarged peripheral lymph nodes were sampled (submandibular, prescapular, and popliteal). The aspirate was then spread on a microscope slide, dried, and stained with modified May-Grunwald-Giemsa (RAL 555, RAL DIAGNOSTICS, Montesquieu site, Martillac, France). All these samples were transferred to the National Reference Laboratory for Leishmaniasis at the National Institute of Hygiene, Rabat, Morocco, for serological (serum samples) and/or molecular-based analysis (fine needle aspirate of lymph nodes).

A complete blood cell count (CBC) (ProCyte DX, IDEXX Laboratories) and a serum biochemistry profile (Catalyst One, IDEXX Laboratories) were performed for 16 and 11 dogs, respectively, due to financial restraints. Medical imaging and dermatological tests were performed on 9 and 3 dogs, respectively. These two tests were used only when needed.

### 2.4. Necropsy of One Dog

A thorough necropsy was performed, according to standard procedures [20], on a 3-year-old pointer *Leishmania*-positive dog that was displaying severe clinical signs, after his owner elected euthanasia. Tissues samples were collected from the liver, kidneys, and lymph nodes and fixed in 10% neutral buffered formalin (10%-NBF) for histopathological examination. Fixed tissues were then processed according to paraffin embedding standard techniques. Briefly, the tissues were dehydrated in alcohol, cleared in toluene, and embedded in paraffin wax. 5 *µ*m thick sections were performed and stained with hematoxylin and eosin (H&E) and examined under a light microscope.

### 2.5. Sample Analysis

#### 2.5.1. Serological Diagnosis

The rapid immunochromatographic strip assay Kalazar Detect Canine Rapid Test (InBios, USA; http://www.InBios.com) was used in this study for the qualitative detection of antibodies against *L. Infantum*, according to the manufacturer's instructions.

#### 2.5.2. Molecular Diagnosis

(1*) DNA Extraction.* DNA was extracted using Qiagen Blood and Tissue kit (Hilden, Germany) according to the manufacturer's instructions. All slides were covered with 200 *µ*l of lysis buffer (ATL). After scraping, the smears were completely removed, and the mixture was transferred to a 1.5 ml reaction tube. A volume of 20 *μ*l of proteinase K was added and incubated between 2 and 4 hours at 56°C, and then 200 *µ*l of binding buffer (AL) was added and the tube was incubated again at 56°C for 10 min. After adding 200 *μ*l of ethanol, the lysate was subjected to two successive washings with a volume of 500 *μ*l each. The DNA was resuspended in 50 *µ*l of elution buffer (TE) and stored at 4°C.

(2*) Real-Time PCR Analysis.* A real-time PCR was performed for the detection of *L. infantum* species using the primers (5′- GGTTAGCCGATGGTGGTCTT -3′) and (5′-GCTATATCATATGTCCAAGCAC TTACCT -3′), as well as the TaqMan MGB 6-FAM dye-labelled probe: 5′- ACCACCTAAGGTCAACCC -3′, as previously described [21].

PCR reactions were performed with ABI TaqMan Universal PCR Master Mix (2X), 900 nM (each) forward and reverse primers, and 250 nM probe. The run consisted of a hold at 95°C for 10 min followed by 40 temperature cycles of 95°C for 15 s and 60°C for 1 min [22]. All assays were carried out with a negative control (DNase-Free Water) and a positive control included in each run.

## 3. Results

Among the 96 included dogs, 32 (33.3%) were positive for *L. infantum* by either serology alone (9/32, 28.1%), qPCR alone (2/32, 6.2%), or both (21/32, 65.6%).

The majority of the positive dogs (29/32 dogs, 90.6%) came from urban areas of northwestern Morocco, including Rabat (*n* = 21), Benslimane (*n* = 2), and Casablanca (*n* = 2), as well as one from each of the following cities: Jorf Al Melha, Rommani, Kenitra, and Fes. The remaining 3 dogs, (9.3%), were from rural areas (Khemisset, Shoul, and Ait Ichou). 20 dogs (62.5%) were males and 12 (37.5%) were females. Ages ranged from 2 to 14 years, with a median of 5 years. 21 (65.6%) were purebreds, 7 (21.8%) were mongrels, and 4 (12.5%) were crossbreds. The two most frequently encountered breeds were German shepherd (*n* = 9) and Braque (*n* = 5). 25 (78.1%) were pet dogs; among them, 5 were exclusively for company and 20 served for various activities (13 guarding, 6 hunting, and 1 farm dog). The remaining dogs were service dogs (3 dogs, 9.3%), shelter (2 dogs, 6.2%), and stray dogs (2 dogs, 6.2%). 19 dogs (59.3%) lived exclusively outdoor and 13 (40.6%) had free access to the outdoor environment. Preventive therapy against ectoparasites was used in 62.5% of dogs (20/32 dogs), mainly using fipronil (14/20 dogs, 70%), and less frequently with other medications including afoxolaner, amitraz, and permethrin (2/20 dogs, 10% each).

Five of the positive dogs (15.62%) were asymptomatic and were presented to the clinic for vaccination. Signs presented by the remaining 27 dogs are listed in Table 1.

**Figure 1:**
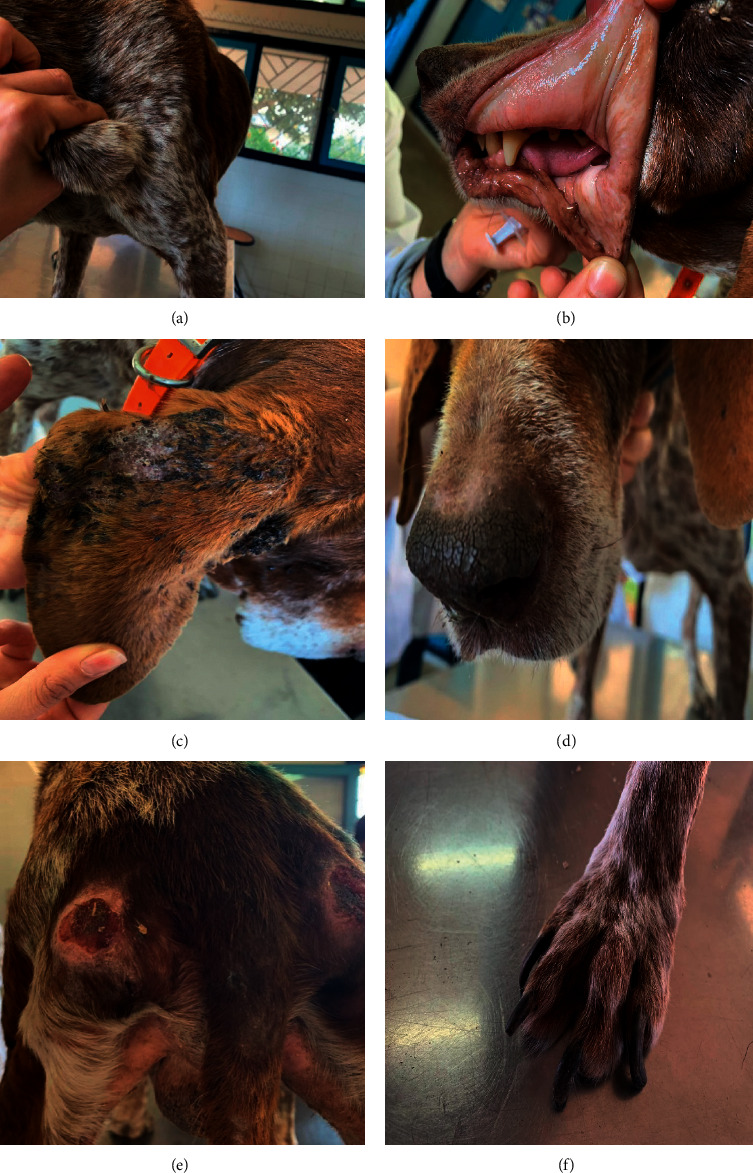
Main clinical signs detected in a *Leishmania*-positive dog. (a) Hypertrophy of the left prescapular lymph node. (b) Pale gingival mucosa. (c) Exfoliative dermatitis (scales, keratinous debris, and alopecia) localized in the right ear. (d) Nasal hyperkeratosis. (e) Bilateral ulcers in pressure points. (f) Onychogryphosis.

General, cutaneous, and ophthalmological disorders were detected in 27 (84.3%), 21 (65.6%), and 9 (28.1%) dogs, respectively. General disorders detected included enlarged lymph nodes (26/32, 81%) (Figure 1(a)), hyperthermia (9/32, 28.1%) defined as body temperature higher than 39.2°C (39.3°C to 40.8°C), pale mucous membranes (6/32, 18.7%), or congested mucous membranes (6/32, 18.7%) (Figure 1(b)). Polyadenomegaly was more frequently detected (*n* = 13, 40.6%) than enlargement of two pairs (*n* = 8, 25.0%) or a single pair (*n* = 5, 15.6%) of lymph nodes.

Generalized, multifocal, or focal skin lesions were noticed in 21 dogs (Table 1). 17 dogs (81%) had more than one lesion at the first consultation. Exfoliative dermatitis (Figure 1(c)) was the most frequently detected one followed by hyperkeratosis (Figure 1(d)) and ulcerative dermatitis (Figure 1(e)). Onychogryphosis was found in 6 dogs (28.5%) (Figure 1(f)).

**Figure 2:**
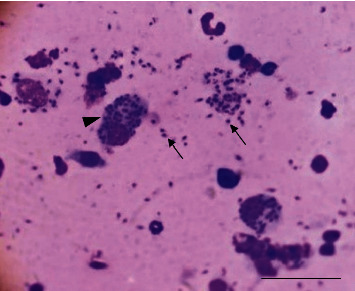
Microscopic examination of a fine needle aspirate of a cutaneous nodule from a *Leishmania*-positive dog (modified MGG) revealing intramacrophages (arrowhead) and extramacrophages (arrows) *Leishmania infantum* amastigotes. Bar = 60 *µ*m.

Aspiration cytology of a skin nodule in one dog revealed multiple free and phagocytized *Leishmania* amastigotes within macrophages leading to the diagnosis of cutaneous *Leishmania* granuloma (Figure 2).

Ophthalmological disorders were detected in 9 dogs and included blepharitis, conjunctivitis, and anterior uveitis in 5 (55.5%), 4 (44.4%), and 2 (22.2%) dogs, respectively. Abdominal ultrasound was performed whenever an abnormality was detected on physical examination and abdominal palpation (pain, mass, and organomegaly) or if complaints suggesting lesions of intra-abdominal organs (vomiting, diarrhea, constipation, melena, and hematuria) were recorded in the history of the dog. Five dogs among the nine for which an abdominal ultrasound was performed (55%) showed evidence of splenomegaly and the remaining four dogs did not show any abnormality.

**Figure 3:**
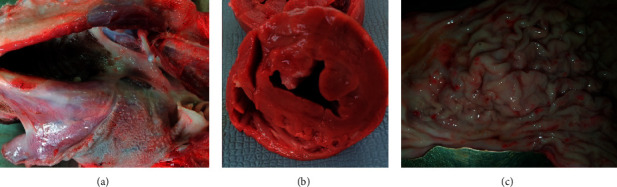
Main postmortem macroscopic lesions detected in a *Leishmania*-positive dog. (a) Ulcerative lesions at the edges of the tongue. (b) Transverse section of the heart showing a hypertrophy of the left ventricle and atrophy and dilatation of the right one. (c) Mucous membrane of the stomach of a *Leishmania*-positive dog, showing petechiae and hemorrhagic suffusions.

**Figure 4:**
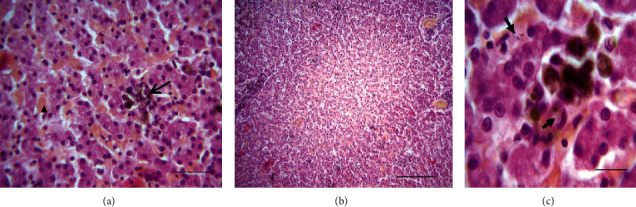
Histologic sections of the liver of a *Leishmania*-positive dog. (a) Moderate congestion of sinusoid capillaries (arrowheads). Agglomerate of hyperplastic Kupffer cells (arrow) showing hemosiderin phagocytosis in sinusoid capillaries, H&E, bar = 90 *µ*m. (b) Moderate centrilobular passive congestions, H&E, bar = 200 *µ*m. (c) Multiple intracytoplasmic bluish inclusions of *Leishmania* spp. amastigotes (arrows) within Kupffer cells. H&E, bar = 40 *µ*m.

**Figure 5:**
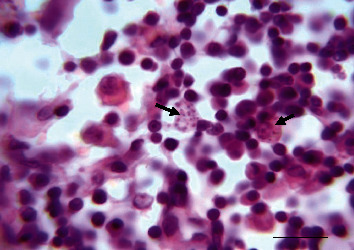
Histologic section of prescapular lymph node of a *Leishmania*-positive dog showing multiple intracytoplasmic bluish inclusions of *Leishmania* spp. amastigotes within macrophages (arrow), H&E, bar = 45 *µ*m.

**Figure 6:**
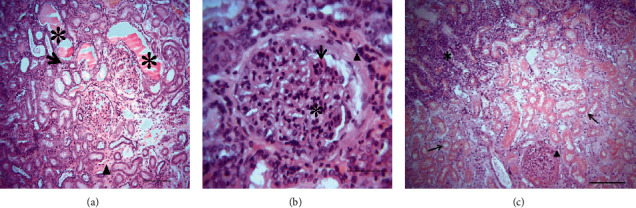
Histologic sections of the kidney of a *Leishmania*-positive dog with suspected membranoproliferative glomerulonephritis. (a) Mild interstitial fibrosis (arrowhead), as well as infiltration by mononuclear cells (arrow). Lumen of renal tubules filled with eosinophilic proteinaceous casts (asterisk), H&E, bar = 200 *µ*m. (b) Hypercellular glomerulus (asterisk), thickening of glomerular basement membrane (arrow), fibrotic outer Bowman's capsule (arrowhead), H&E, bar = 70 *µ*m. (c) Margins of a renal infarct (asterisk). Coagulative necrosis of tubules (arrows) and glomeruli (asterisk), H&E, bar = 200 *µ*m.

At the necropsy performed on the euthanized dog, multiple macroscopic lesions were detected including pale ocular and oral mucosa and subcutaneous connective tissue (Supplementary Figure 1), enlargement, and greenish to eosinophilic appearance of the superficial lymph nodes (Supplementary Figure 2). The oral cavity showed ulcerative lesions at the edges of the tongue (Figure 3(a)). The heart revealed a rounded apex, hypertrophy of the left ventricle, and atrophy and dilatation of the right one (Figure 3(b)). In the liver, pathological findings consisted of multiple discolored areas and rounded edges due to hepatomegaly (Supplementary Figure 3). Both kidneys were slightly pale with irregular grainy surface and hard in consistency and presented depressed infarcted areas localized to the cortex (Supplementary Figure 4). The mucous membrane of the stomach presented petechiae and hemorrhagic suffusions (Figure 3(c)). No gross changes were detected in other organs. Microscopically, the liver parenchyma presented a centrolobular vasodilatation and stasis, multiple foci of Kupffer cell hyperplasia within sinusoid capillaries, and hemosiderin phagocytosis providing evidence of a hemolytic process (Figures 4(a) and 4(b)). Multiple intraphagocytic inclusions of *Leishmania infantum* amastigotes were identified in the cytoplasm of both Kupffer cells and macrophages in the liver (Figure 4(c)) and the prescapular lymph node (Figure 5), respectively. The kidneys showed subchronic membranoproliferative glomerulonephritis (Figures 6(a) and 6(b)) as indicated by hypercellular glomeruli and thickening of their basement membrane. The outer Bowman's capsule of some glomeruli was fibrotic and renal interstitial tissue was diffusely widened by a mild-to-moderate mononuclear cell inflammatory infiltrate (lymphocytes, plasma cells, and macrophages), as well as fibrosis. Amorphous and eosinophilic protein casts were seen within the lumen of renal tubules evoking proteinuria. In those depressed cortical areas noticed at the macroscopic examination, there was a severe coagulative necrosis of the tubules and glomeruli along with intravascular thrombi, which is consistent with the diagnosis of renal infarcts (Figure 6(c)).

Results of CBC and biochemistry profile were available for 16 and 11 *Leishmani*a-positive dogs, respectively. Anemia (11/16, 68.7%) and thrombocytopenia (5/16, 31.2%) were the most frequent changes detected in those dogs. Anemia was nonregenerative, normocytic, and normochromic, with hematocrits, hemoglobin concentrations, and red blood cell counts ranging from 20.1 to 35.8% (RI: 37.3–61.7%), from 7.1 to 12.2 g/dl (RI: 13.1–20.5 g/dl), and from 2.99 to 5.59 M/*μ*l (RI: 5.65–8.87 M/*μ*l), respectively. Thrombocytopenia (5/16, 31%) was characterized as mild to severe, ranging from 0 to 140 k/*μ*l (RI: 148–484 k/*μ*l).

Hyperproteinemia due to hyperglobulinemia was the only abnormality recorded in 8 (72.7%) *Leishmania*-positive dogs among the 11 ones for which a biochemistry profile was performed.

## 4. Discussion

Visceral leishmaniasis (VL), caused by *Leishmania infantum*, is, according to the World Health Organization (WHO), one of the most important neglected tropical diseases [23]. Every year, around 0.5 million people are infected by VL [24]. The disease results in death if left untreated and shows a mortality rate of 6% in children [24]. VL is attracting more interest in Morocco because of the infant mortality rate that varies between 2 and 8% [25]. In addition, a clear increase in both the incidence and the geographic distribution of this disease has been observed recently. During the period between 1999 and 2014, the annual incidence rate of VL reported in Morocco was 0.4 per 100,000 inhabitants [26].

As they are the main reservoir for VL caused by *L. infantum*, dogs play a critical role in the transmission of the disease to humans. In addition, dogs could contribute to the introduction or even the dissemination of the disease in nonendemic countries [24]. Indeed, a recent report has described a *Leishmania*-positive symptomatic Moroccan dog introduced in Quebec, Canada [27]. Similarly, several studies have described an expansion of CanL from southern to northern regions of Europe [28–30].

This important epidemiological role played by dogs in the parasitic life cycle emphasizes the need for improving the knowledge on CanL, especially in Morocco. Indeed, even if some previous studies reported clinical signs of CanL in Moroccan dogs [6, 17, 31], to our knowledge, this is the first study that aimed to examine, describe, and discuss clinical signs of CanL in Moroccan dogs that presented to a veterinary health structure and were coming mainly from urban areas. This increased prevalence of CanL in urban communes can be explained by the impact of urbanization and the concentration of the population. It is of interest to note that urbanization and especially periurbanization cause the rapid expansion of large agglomerations which are generally overpopulated and provide inadequate housing and poor sanitation facilities, hence the risk of the proliferation of vectors and reservoirs of the parasite and consequently the increase of leishmaniasis cases [32].

Among dogs assessed in the present study, 33.3% were positive for *L. infantum* by either serology alone, qPCR alone, or both. The serological and the molecular tests used confer sensitivity values of 95.8% and 100%, respectively, and specificity values of 100% and 96.4%, respectively [33, 34].

The results showed that older dogs, males, purebreds, and dogs with considerable exposure to outdoor environments without effective ectoparasite prevention were more frequently *Leishmania*-positive. Several studies had reported that older dogs are more frequently infected than younger ones [35–39]. Cortes et al. [38] found that dogs below 2 years of age were rarely infected, while most infected dogs were between 5 and 8 years of age. Cardoso et al. [37] noticed that older dogs (9–11 years old) are twice more infected than younger dogs (0–2 years old). This could be justified by the long incubation period of the disease (extending up to four years) [40]. Furthermore, repetitive exposure to infected sandflies in older animals could be an additional predisposing factor [38]. On the other hand, the seroprevalence follows a bimodal age distribution, with a peak under the age of three years and a second one at the age of 8–10 years [41–43]. This distribution has been assigned to the dog sensitivity. Sensitive dogs will develop the disease at an early age, and latent infections will not be activated until they are older and their immune system declines or they develop comorbidities [42, 43]. Additionally, infection in young dogs could be attributed to a potential congenital contamination [44], although this mode of transmission is still controversial [45].

In this study, males were more frequently positive than females. Although gender is not considered as a determinant factor of leishmaniasis [36, 38, 41], some studies reported the same observation [35, 39, 42, 46]. Miranda et al. [42] assigned this to the higher errant behavior of male dogs. Dogs staying outside the house are likely to be exposed to sandflies longer and then show a higher risk of infection with *Leishmania* spp. [43].

Purebreds seem to be more infected than mixed breeds [35, 36, 39]. According to Cortes et al. [38], being mongrel could be a protective factor, while being a pure exotic breed could be a risk factor. Some studies have associated specific breeds with a higher susceptibility to the disease [47, 48], while others did not confirm such relationship [41, 49]. In this survey, German shepherd (*n* = 9) and Braque (*n* = 5) were the most frequent purebreds included; however, this could be attributed to the significant role that these breeds play in guarding and hunting in Morocco, respectively.

Products containing synthetic pyrethroids, permethrin, or deltamethrin are available for the prevention of canine leishmaniasis [50]. These products are commercialized in a spot-on or collar formulation [50]. However, in this study, most dog owners (18/20, 90.0%) used insecticides free of pyrethroids. Only two used permethrin but not regularly, which can explain their infection. This low rate of prevention using pyrethroids could be justified by the low availability of these products in Morocco. Actually, during the period of sampling, only one insecticide containing permethrin (associated with fipronil) was commercialized in the country. A second product (deltamethrin-impregnated collar) started to be commercialized from July 2020. In addition, dog owners are not completely aware of the necessity of using such products and may also be discouraged by the relatively expensive cost of their regular use. However, the appropriate use of insecticides remains essential for the protection of dogs living in the Mediterranean Basin [51].

Since the dogs of this study were mostly selected based on clinical signs typical of CanL or epidemiological factors associated with increased risk of acquiring this infection, most sampled dogs were symptomatic, which explains the low prevalence of asymptomatic dogs in our results (5 dogs, 15.62%). However, many studies have shown that between 35% and 60% of infected dogs are asymptomatic [7, 47, 52], which could be due to the fact that the disease was at an initial stage [47] or due to the effectiveness of the immune response of the host (Th1-type) [53]. Those asymptomatic dogs play an important epidemiological role, since they are potentially infectious to sandflies [54]. Therefore, a proper assessment of the infection status of dogs living in endemic areas could be of a great benefit to the control of the disease.

Similar to previous literature, skin lesions, weight loss, exercise intolerance, decreased appetite or anorexia [6, 17, 31, 35, 36], and lymphadenomegaly [17, 31, 35, 36, 38, 46, 55] were the most commonly noticed clinical signs. CanL-associated lymphadenomegaly is reported to be the result of the mononuclear-phagocytic system reaction to blood-borne Leishmania spp. antigens [36]. Cytologically, Mylonakis et al. [56] found that 59.4% of dogs with clinical leishmaniosis had abnormal lymph node cytology and mostly involved lymphoid hyperplasia. However, histiocytic lymphadenitis, neutrophilic lymphadenitis, and eosinophilic lymphadenitis have also been reported [56]. Generalized lymphadenomegaly was more frequent in our study than the hypertrophy of one pair of lymph node, which is in accordance with the findings of a previous study [46]. Interestingly, when lymphadenomegaly was symmetrical, the prescapular and popliteal lymph nodes were the most frequently enlarged as reported in previous surveys [31, 46]. According to Ciaramella et al. [46], the more frequent hypertrophy of the prescapular lymph nodes could be attributed to the connection between these nodes and the lymphatic vessels draining the cranial regions which frequently display the most severe skin lesions.

As previously described [36, 38], we have frequently detected skin lesions in *Leishmania*-positive dogs mostly expressed as exfoliative dermatitis [36, 39, 46, 55]. This skin disorder is characterized mainly by excessive scaling and could be associated with alopecia, pruritus, “rancid fat” odor, accumulation of keratinous debris, and hair follicles filled with oil and skin cells (comedones) [57]. Exfoliative dermatitis has been associated with an effective immune response in CanL [39, 58]. Hyperkeratosis was also frequently detected in our study, mostly located on the dorsal part of the nose, and was reported with prevalence ranging from 4.5% to 18.8% in other studies [36, 55]. Mucocutaneous ulcerations are relatively prevalent in CanL [36, 46]. In the surveys conducted by Koutinas et al. [36] and Ciaramella et al. [46], ulcerative lesions represented the second most commonly detected skin disorder and were noted in 34.4% and 40% of the dogs, respectively. However, ulcers were less frequently recorded by other authors [35, 39]. In this study, ulcerations were rather common with prevalence of 28.5%. As previously described [36, 39, 46, 55], ulcerative lesions were most frequently located in pressure points and pinnae. Skin nodules were detected in 3 out of the 21 dogs with cutaneous lesions (14.2%). This prevalence is higher than what was reported in previous studies, where skin nodules were detected in 11.0% [39], 4.5% [55], and 2.3% [36] of the dogs with cutaneous manifestations. This nodular form has been correlated with a weak immune response by some authors [36, 58], while others associated it with a favorable immune response [39, 59]. In this study, nodules were detected in 3 different breeds (*Dogo canario*, *Canis vulgaris*, and a mixed German Shepherd breed), which is in line with what was previously described [36, 46]. Another report showed that Boxers were more susceptible to develop skin nodules [60]. Although the most common lesions associated with CanL are exfoliative dermatitis and hyperkeratosis, other forms could also be noticed (e.g., nodules, ulcers, and pododermatitis). These observations make it essential for veterinarians to include leishmaniasis in the differential diagnosis of dogs presenting these lesions in endemic areas, especially if they are refractory to previous treatments.

Ocular conditions were reported in CanL with prevalence ranging from 16% to 71.1% [46, 61], which is in accordance with our findings (28%). Reported ocular lesions included conjunctivitis, keratoconjunctivitis sicca (KCS) [17, 36, 46, 62], uveitis [36, 46, 60, 62], blepharitis, and scleritis [36, 60–62]. These ocular lesions could be related to an immunoglobulin G (IgG) deposition or to the infiltration of plasma cells and macrophages containing the parasites in various ocular compartments [63].

Five (55.5%) of the nine dogs for whom abdominal ultrasound was performed showed splenomegaly. This is in line with the findings of a recent study where splenomegaly was the most common abdominal ultrasound abnormality associated with CanL [64].

At postmortem examination of the euthanized dog, oral ulcers were detected. Such changes were previously described [59, 65, 66] and could be caused by the action of the parasite [67] or could be the result of a potential renal failure (uremic state) [65, 66]. Seemingly, the petechiae and hemorrhagic suffusions detected in the stomach of the same dog could be the result of hemostatic disorders usually associated with CanL [67] or due to a possible uremic state. Histopathology of the kidney revealed a renal infarction and subchronic membranoproliferative glomerulonephritis. Glomerulonephritis (either membranous, membranoproliferative, mesangioproliferative, or a combination) is a common complication of CanL [68, 69] that can lead to renal failure (azotemia and proteinuria) [36, 46]. This renal lesion worsens the prognosis of the disease [70] and makes the treatment more challenging [71]. Renal infarcts noticed in this dog were associated with intravascular thrombi which may be highly linked to disseminated intravascular coagulation (DIC). Indeed, DIC and hemostasis abnormalities have been previously reported to occur in *Leishmania*-affected dogs [72]. This dog also presented cardiac abnormalities (dilatation of the right ventricle and hypertrophy of the left one) which may be related to increased blood pressure due to the glomerulonephritis. The passive congestion of the dog's liver as shown by the centrolobular vasodilation can be linked to a right heart failure. Hyperplasia and hypertrophy of the Kupffer cells were also noticed. Indeed, this later change was previously described in a hamster subjected to an experimental infection with *Leishmania donovani* [73]. Moreover, histology revealed *Leishmania infantum* amastigotes in both the liver and the left prescapular lymph node parenchyma, which can be attributed to the extensive distribution of the parasite throughout the body (liver, lymph nodes, spleen, bone marrow, kidney, and skin) [15].

Unfortunately, one of the limitations of our study is that urinalysis was not performed, and complete blood cell count and biochemistry profile were not done for all studied dogs due to economic reasons. Thus, for future studies, we recommend evaluating the urinary, hematological, and biochemical changes in symptomatic and asymptomatic patients. It could also be interesting to evaluate, for the first time in Morocco, possible coinfections between *L. infantum* and other vector-borne pathogens (VBPs) in dogs, such as *Dirofilaria immitis*, *Ehrlichia canis*, *Anaplasma* spp., and *Babesia* spp., which are known to be present in Morocco [74]. VBPs usually cause a similar clinical picture and coinfections may lead to atypical clinical signs making the diagnosis and the management challenging for veterinarians [75]. Furthermore, various countries from the Mediterranean region, with environment and climate conditions similar to Morocco, have reported such coinfections, hence the interest of investigating this eventuality in Moroccan dogs [75].

Finally, it is important to mention that, to control leishmaniasis, the WHO proposes various key strategies including early diagnosis and effective prompt treatment, vector control, effective disease surveillance, control of animal reservoir hosts, social mobilization, and strengthening partnerships [76].

However, the diagnosis of canine leishmaniasis is challenging because of its nonspecific clinical manifestations and the lack of confirmation of diagnostic tests and their nonavailability to veterinarians in Morocco. Indeed, currently no laboratory in Morocco provides quantitative serology or molecular tests for the diagnosis of CanL. Only a rapid test based on a qualitative serological method is available (Snap *Leishmania*, IDEXX Laboratories). Once a dog is confirmed to be positive, the owners are informed about the severity and the zoonotic aspect of the disease and the decision to treat or euthanize the animal remains the owner's choice, since there is no legislation in Morocco that regulates the management of leishmaniasis in dogs. Moreover, there is no scientific evidence that culling seropositive dogs could reduce the incidence of VL [77]. The treatment protocol is based on the use of allopurinol associated with either meglumine antimoniate or miltefosine and a regular use of effective repellents containing pyrethroids as a prophylactic measure [78]. However, when owners opt for the treatment, they are confronted with the lack of availability of both meglumine antimoniate (reserved for the treatment of human leishmaniasis in hospitals) and miltefosine in the country. In this case, owners usually have to import the relevant drugs from a European country, which is unfortunately not feasible for everyone. In this study, only 13 dog owners had the willingness and the financial ability to comply with the treatment protocol. Eleven dogs were treated with the association of allopurinol (Zyloric: 10 mg/kg BID) and miltefosine (Milteforan: 2 mg/kg SID), and 2 dogs received only allopurinol (because the owners could not afford to purchase miltefosine from a European country). In addition, dog owners were advised to ensure a constant use of insecticides containing permethrin. Nine of the treated dogs were monitored after treatment for at least one month, and all showed a complete clinical recovery.

## 5. Conclusion

The results of this study suggest that purebreds, adults, and dogs with considerable exposure to outdoor environments are more susceptible to developing CanL. A substantial proportion of positive dogs were asymptomatic, constituting an epidemiological risk, since they are infectious to sandflies. In symptomatic dogs, the main detected clinical signs were skin lesions, weight loss, exercise intolerance, decreased appetite or anorexia, and lymphadenomegaly. Macroscopic and microscopic lesions of the euthanized dog involved mainly the tongue, lymph nodes, stomach, liver, and kidneys. A helpful description and projection of the main clinical signs and macroscopic and microscopic lesions observed in the studied dogs are available in this paper.

These results were consistent with the literature, confirming the vague and nonspecific clinical manifestations of CanL. For this reason, we encourage veterinarians to systematically test suspected dogs and to screen at-risk dogs for leishmaniasis on a yearly basis. On the other hand, dog owners should be made aware of this zoonosis and the main prevention measures so that they can contribute to the control of the disease in humans and in animals. Finally, a collaboration of physicians and veterinarians following the One Health approach is essential to identify and address zoonotic diseases in both humans and animals and hence contribute to a more effective control/prevention.

## Figures and Tables

**Table 1:** Main clinical signs and complaints mentioned by the owners for the 27 dogs with CanL.

Clinical signs and owners' complaints		Number of dogs	Percentage
Weight loss		19	59.4
Exercise intolerance		18	56.2
Appetite disorders	Anorexia	9	28.1
	Hyporexia	5	15.6
Exfoliative dermatitis	Diffuse	5	15.7
	Multifocal	4	12.5
	Focal	4	12.5
Hyperkeratosis	Diffuse	3	9.3
	Focal (nasal)	5	15.7
	Multifocal	1	3.1
Ocular signs		9	28.1
Gastrointestinal disorders		6	18.7
Focal ulcer		5	15.7
Focal nodule		3	9.3
Epistaxis		3	9.3
Pododermatitis		2	6.2
Focal necrosis		1	3.1

## Data Availability

The data used to support the findings of this study are included within the article.

## Supplementary Materials

Supplementary Materials Supplementary Figure 1: pallor detected at necropsy of a leishmania-positive dog. A: pale ocular mucosa. B: pale oral mucosa. C: pale subcutaneous connective tissue. Supplementary Figure 2: section of the prescapular lymph node of a *Leishmania*-positive dog showing a greenish to eosinophilic appearance. Supplementary Figure 3: congested and slightly enlarged liver of a *Leishmania*-positive dog with the presence of multiple foci of discoloration. Supplementary Figure 4: kidneys of a *Leishmania*-positive dog showing a grainy surface and pale depressed areas, localized to the cortex with a congestive-hemorrhagic margin (arrows).
