# Supplementary figures and images for: Canine Leishmaniasis in Morocco: A Descriptive Prospective Clinical Study
Source: Vet Med Int. 2021 Sep 6;2021:6304127. doi: 10.1155/2021/6304127 (PMC8440073; doi:10.1155/2021/6304127)

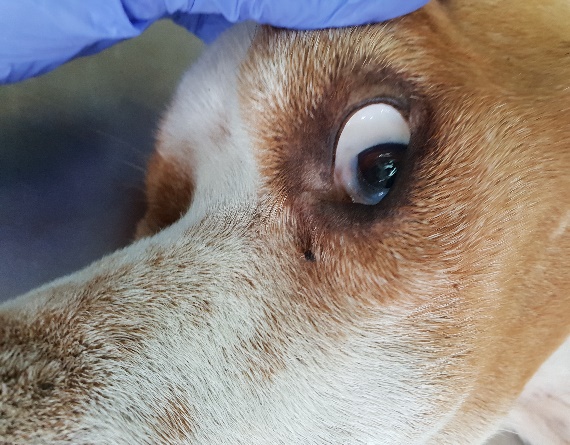

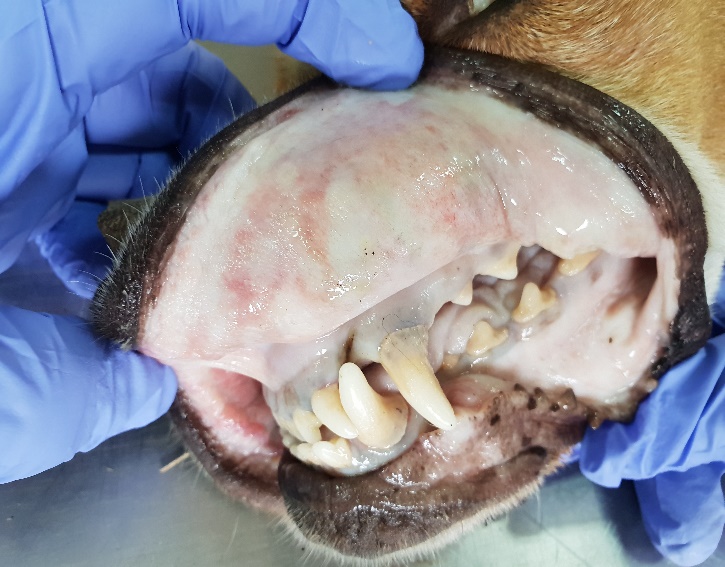

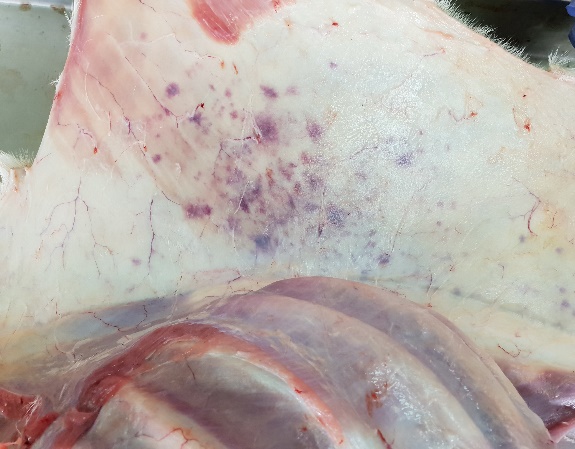


**A**

**B**

**C**

Supplement: Supplementary Materials — Supplementary Figure 1: pallor detected at necropsy of a leishmania-positive dog. A: pale ocular mucosa. B: pale oral mucosa. C: pale subcutaneous connective tissue. Supplementary Figure 2: section of the prescapular lymph node of a Leishmania-positive dog showing a greenish to eosinophilic appearance. Supplementary Figure 3: congested and slightly enlarged liver of a Leishmania-positive dog with the presence of multiple foci of discoloration. Supplementary Figure 4: kidneys of a Leishmania-positive dog showing a grainy surface and pale depressed areas, localized to the cortex with a congestive-hemorrhagic margin (arrows). [file 6304127.f1.zip › 6304127.f1/supplemental figure 1.docx]

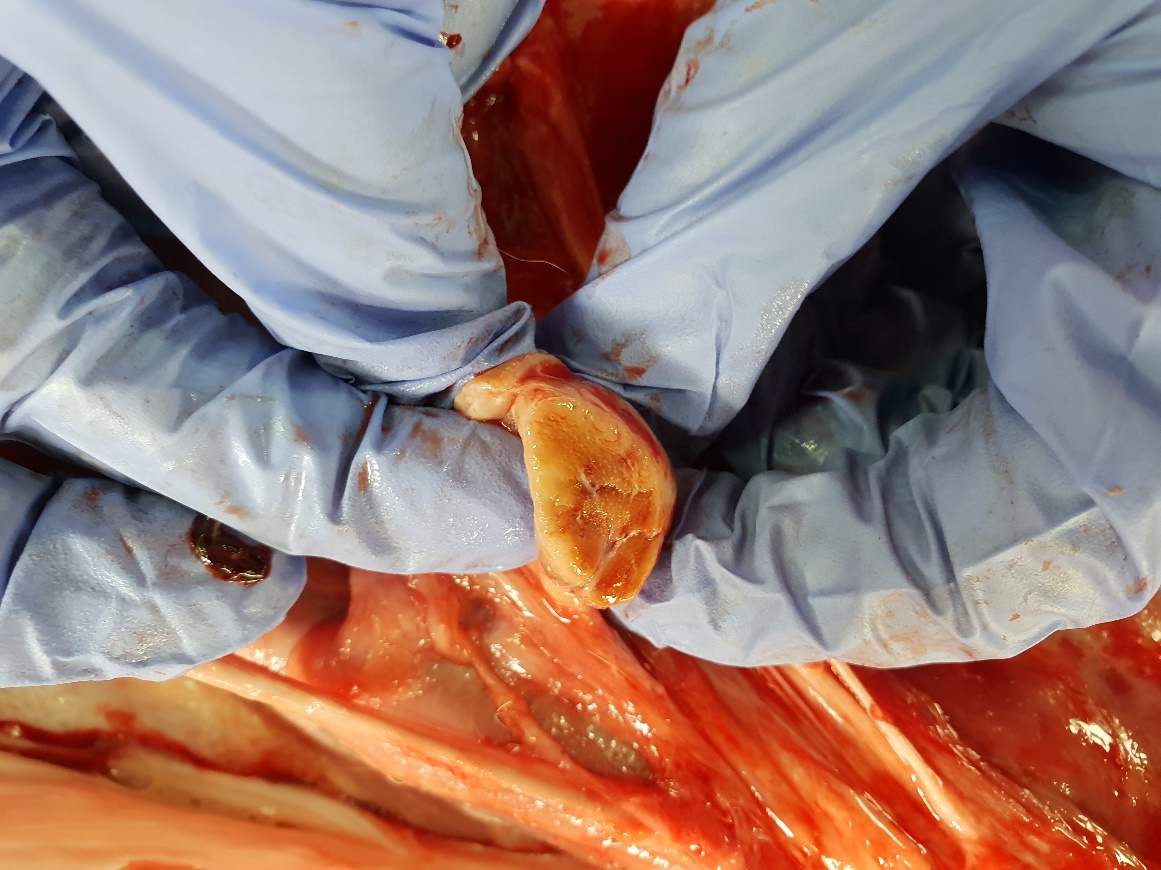

Supplement: Supplementary Materials — Supplementary Figure 1: pallor detected at necropsy of a leishmania-positive dog. A: pale ocular mucosa. B: pale oral mucosa. C: pale subcutaneous connective tissue. Supplementary Figure 2: section of the prescapular lymph node of a Leishmania-positive dog showing a greenish to eosinophilic appearance. Supplementary Figure 3: congested and slightly enlarged liver of a Leishmania-positive dog with the presence of multiple foci of discoloration. Supplementary Figure 4: kidneys of a Leishmania-positive dog showing a grainy surface and pale depressed areas, localized to the cortex with a congestive-hemorrhagic margin (arrows). [file 6304127.f1.zip › 6304127.f1/supplemental figure 2.docx]

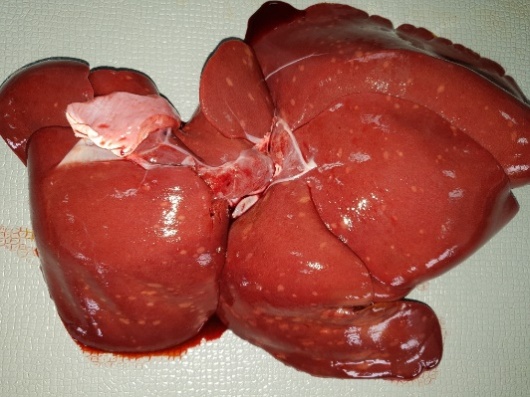

Supplement: Supplementary Materials — Supplementary Figure 1: pallor detected at necropsy of a leishmania-positive dog. A: pale ocular mucosa. B: pale oral mucosa. C: pale subcutaneous connective tissue. Supplementary Figure 2: section of the prescapular lymph node of a Leishmania-positive dog showing a greenish to eosinophilic appearance. Supplementary Figure 3: congested and slightly enlarged liver of a Leishmania-positive dog with the presence of multiple foci of discoloration. Supplementary Figure 4: kidneys of a Leishmania-positive dog showing a grainy surface and pale depressed areas, localized to the cortex with a congestive-hemorrhagic margin (arrows). [file 6304127.f1.zip › 6304127.f1/supplemental figure 3.docx]

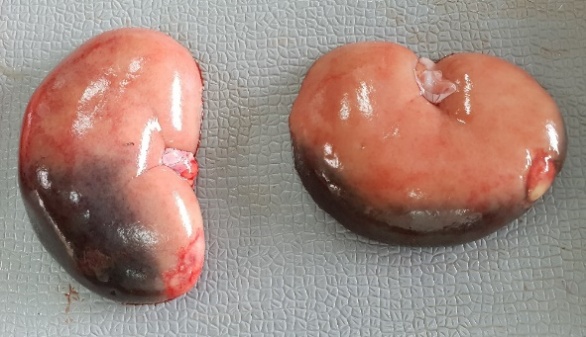

Supplement: Supplementary Materials — Supplementary Figure 1: pallor detected at necropsy of a leishmania-positive dog. A: pale ocular mucosa. B: pale oral mucosa. C: pale subcutaneous connective tissue. Supplementary Figure 2: section of the prescapular lymph node of a Leishmania-positive dog showing a greenish to eosinophilic appearance. Supplementary Figure 3: congested and slightly enlarged liver of a Leishmania-positive dog with the presence of multiple foci of discoloration. Supplementary Figure 4: kidneys of a Leishmania-positive dog showing a grainy surface and pale depressed areas, localized to the cortex with a congestive-hemorrhagic margin (arrows). [file 6304127.f1.zip › 6304127.f1/supplemental figure 4.docx]
